# Development of a core outcome set for clinical trials in basal cell carcinoma: study protocol for a systematic review of the literature and identification of a core outcome set using a Delphi survey

**DOI:** 10.1186/s13063-017-2244-5

**Published:** 2017-10-23

**Authors:** Daniel I. Schlessinger, Sanjana Iyengar, Arianna F. Yanes, Jake M. Lazaroff, Victoria Godinez-Puig, Brian R. Chen, Anastasia O. Kurta, Jill K. Henley, Sarah G. Chiren, Karina C. Furlan, Jochen Schmitt, Stefanie Deckert, Emily Poon, Joseph F. Sobanko, Todd V. Cartee, Murad Alam, Ian A. Maher

**Affiliations:** 1 Department of Dermatology, Feinberg School of Medicine, Northwestern University, 676 N. St. Clair St., Ste 1600, Chicago, IL 60611 USA; 2 Department of Dermatology, Saint Louis University School of Medicine, St. Louis, MO USA; 3 Department of Dermatology, University of Pennsylvania, Philadelphia, PA USA; 4 Centre for Evidence-Based Healthcare, Medizinische Fakultät Carl Gustav Carus, TU Dresden, Dresden, Germany; 5 Division of Dermatologic Surgery, University of Pennsylvania, Philadelphia, PA USA; 6 Department of Dermatology, Penn State Hershey Dermatology, Hershey, PA USA; 7 Department of Otolaryngology, Feinberg School of Medicine, Northwestern University, Chicago, IL USA; 8 Department of Surgery, Feinberg School of Medicine, Northwestern University, Chicago, IL USA

**Keywords:** Core outcome set, Delphi, Consensus, Stakeholders, Basal cell carcinoma, Systematic review

## Abstract

**Background:**

Basal cell carcinoma is the most common skin cancer worldwide. Treatment options include both surgical and topical modalities. Although risk of metastasis is low, basal cell carcinoma can be invasive and infiltrate important underlying structures such as bone or cartilage. While many clinical trials examining therapies for basal cell carcinoma exist, the lack of consensus in outcome reporting across all trials poses a concern. Proper evaluation and comparison of treatment modalities is challenging. In order to address the inconsistencies present, this project aims to determine a core set of outcomes which should be evaluated in all clinical trials of basal cell carcinoma.

**Methods/design:**

Outcomes will be extracted over four phases: (1) a systematic literature review, (2) patient interviews, (3) other published sources, and (4) stakeholder involvement. Potential outcomes will then be examined by the Steering Committee, who may add or remove outcomes. The Delphi process will then be performed to condense the list of outcomes generated. Two rounds of Delphi surveys will be performed with two groups of participants – physicians and patients. A consensus meeting with relevant stakeholders will be conducted after the Delphi exercise to further select outcomes, taking into account participant scores. By the end of the meeting, members will vote and decide on a final recommended set of core outcomes. For the duration of the study, we will be in collaboration with both the Core Outcome Measures in Effectiveness Trials (COMET) initiative and the Cochrane Skin Group – Core Outcome Set Initiative (CSG-COUSIN).

**Discussion:**

This study aims to develop a core outcome set to guide assessment in clinical trials on basal cell carcinoma. The end-goal is to improve the consistency of outcome reporting and allow proper evaluation of treatment effectiveness.

## Background

Basal cell carcinoma (BCC), which arises from the basal layer of the epidermis, is the most common cancer of the skin [1, 2]. It classically presents as pink or pearly papules or plaques with rolled borders, central crusting, or ulceration [3]. BCC is particularly common in Caucasians, men, older people, and those living close to the equator. Predisposing risk factors for the development of BCC include sun, ultraviolet radiation, arsenic exposure, use of photosensitizing drugs, immunosuppression, and genetic susceptibility [4–6]. BCC can be divided into the following subtypes based on histology: nodular, micronodular, superficial, cystic, morpheaform, and infiltrative [3, 7]. Treatments include standard excision, Mohs micrographic surgery, electrodessication and curettage, radiotherapy or cryotherapy. BCC can be treated topically with 5-fluorouracil, imiquimod, or photodynamic therapy [8, 9]. Although the risk of metastasis is small, BCC can be invasive and may infiltrate underlying nerves, muscle, bone or cartilage [10, 11]. As BCCs commonly occur on the face, its removal can cause considerable cosmetic disfigurement and can affect an individual’s quality of life.

While Cochrane reviews and other systematic reviews have investigated the efficacy of various treatments, there is heterogeneity in outcomes assessed across trials [8]. This inconsistency in outcomes measured poses a concern when evaluating the effects of different interventions. Selective outcome reporting bias, defined as results-based selection of outcomes for publication, is a problem in many clinical trials and affects the conclusions of a significant proportion of systematic reviews [12].

In order to address this concern, specific organizations have been formed. The Core Outcome Measures in Effectiveness Trials Initiative (COMET) brings together researchers interested in developing a standardized set of core outcomes in various health-related fields [13]. A core outcome set (COS) is defined as an agreed minimum set of outcomes that is recommended to be measured and reported in all clinical trials of a given condition or disease. Similarly, another group, the Cochrane Skin Group – Core Outcome Set Initiative (CSG-COUSIN), was created specifically to address COSs in dermatology by examining outcome measures in current research [14, 15]. CSG-COUSIN builds on the experiences of the Harmonizing Outcome Measures for Eczema (HOME) initiative, which also developed a roadmap to guide the process of COS development and implementation [16–21]. Both groups hope to develop standardized, evidence-based COSs which can be utilized in all clinical trials.

While COSs are under development for several dermatologic conditions, work has yet to be done to identify core outcomes specific for BCC. In order to minimize duplication, this study has been registered with the COMET and CSG-COUSIN organizations. Interested researchers may contact us for further information.

### Objective

The aim of this study is to develop an international COS relevant to clinical trials for BCC. The objective will be to determine which outcomes should be measured in clinical trials, as well as method of assessment. These core outcomes suggest the minimum that should be reported in research trials but do not limit other outcomes from being investigated.

## Methods/design

**Fig. 1:**
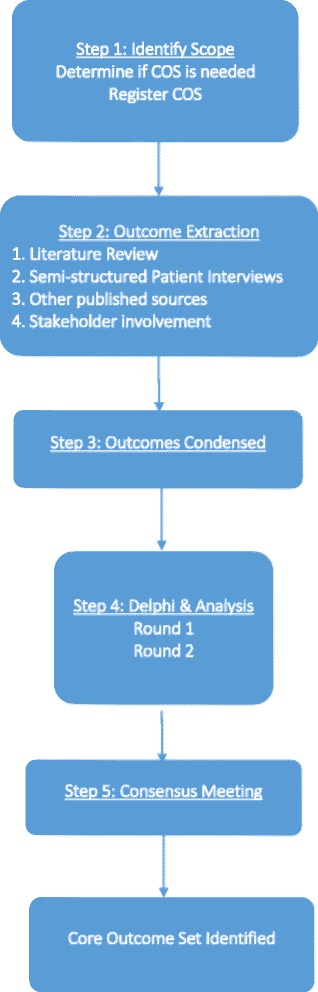
Flowchart of the study design

The development of this COS adheres to the recommendations provided by the COMET and CSG-COUSIN initiatives, with reporting conforming to the SPIRIT (Standard Protocol Items: Recommendations for Interventional Trials) Checklist [13, 21]. This project has been adapted from a previously published protocol [22]. Figure 1 provides a brief overview of our study design (modified from reference [22]).

### Scope of this COS

This COS is intended as the global/international standard for clinical trials examining the efficacy of various treatments for BCC. The COS to be developed will not be population specific and will encompass outcomes from all basal cell therapies.

### Identification of outcomes

Outcomes will be generated over four phases:

1. Systematic literature review: outcomes published in randomized controlled trials and Cochrane reviews will be extracted
2. Patient interviews: interviews will be conducted with patients to determine outcomes valued by patients
3. Other sources: sources, such as clinical trial registries and BCC educational brochures, will be reviewed to ensure that all outcomes have been documented
4. Stakeholders: input from stakeholders will be elicited to provide further insight into outcomes that they would like included

### Literature review

A systematic literature review using PubMed, Medline, and Embase will be conducted using “basal cell carcinoma” and “BCC” as search terms, with queries limited to the title or abstract fields. The systematic review will adhere to the Preferred Reporting Items for Systematic Reviews and Meta-Analyses (PRISMA) guidelines [23]. Included studies will be randomized controlled trials with duplicate studies represented in the various databases included only once. Study characteristics, such as authors, year of publication, source of funding, methodology, number of study centers, treatment comparators, treatment vehicle, inclusion and exclusion criteria, and blinding and randomization status, will be documented. Study design characteristics, such as length of follow-up, treatment duration, results, outcomes, and outcome measures, will be noted. Data will be entered by eight data extractors from four different universities into a spreadsheet. Entries will be periodically reviewed by two senior data extractors for accuracy. The long-list of outcomes extracted from published studies will then be placed into appropriate domains by two Measurement of Priority Outcome Variables in Dermatologic Surgery (IMPROVED) investigators using a forced consensus method. Similar outcomes will be listed only once. Combining and collapsing of outcomes, while necessary, will be performed in moderation to prevent loss of content.

### Patient-centered outcomes

Semi-structured interviews will be conducted to explore patient-identified outcomes. Patients, both in the United States and worldwide, will be recruited from the current patients of practicing physicians and skin cancer advocacy groups via emails and phone calls. Open-ended questions will allow for patient expression of outcomes important to them. According to the research concept to theoretical saturation, approximately 10–15 patients with BCC will be interviewed. This refers to the number of differing opinions required to adequately represent qualitative research findings without further identification of new themes [24]. Past research reveals the requisite number to be 12 or more interviews, with even six interviews identifying most basic themes [25]. A global context will be provided by including participants both in the United States and internationally. Interviews will be audio-recorded, transcribed, and coded to allow complete capturing of outcomes.

#### Additional sources

Examination of other published sources, such as clinical trial registries, Cochrane reviews, pamphlets, and brochures, will be conducted to gather outcomes related to BCC. Additional outcomes extracted will be included in the final list of outcomes.

### Stakeholder involvement

Stakeholders, or those invested in the development of a COS in BCC, will also be included in the decision process (Table 1). Dermatologists, drug and device safety regulators (e.g., US Food and Drugs Administration (FDA), European Medicines Agency (EMA)), pharmacologists, pharmacists, and industry scientists are potential members that can provide input regarding what outcomes they feel should be represented. Nurses, physician assistants, and other health care practitioners may be included as well to enhance further discussion.

**Table 1:** Summary of stakeholder involvement

Key stakeholders
Physicians (*including dermatologists, international providers, physicians of other health care fields*)
Patients
Drug and device safety regulators (e.g., FDA, EMA)
Pharmacologists/pharmacists
Industry scientists
Nurses, physician assistants, or other health care providers

*EMA* European Medicines Agency, *FDA* US Food and Drugs Administration

### Potential outcomes

Outcomes obtained from the steps described above will then be examined by the Steering Committee, composed of four dermatologists: Drs. Murad Alam (Northwestern University), Ian A. Maher (Saint Louis University), Joseph F. Sobanko (University of Pennsylvania), and Todd V. Cartee (Pennsylvania State University). Members may add or remove outcomes prior to the Delphi process. The Steering Committee members will not join in the Delphi process but will be invited to participate in the final consensus meeting.

### Delphi overview

Delphi surveys have been used in prior COS research [26]. The process involves a series of rounds of data collection and analysis to condense the opinions of individuals into a group consensus. Surveys can be conducted online through the use of specialized software. Responses to each round are collected, analyzed, and then redistributed to participants in successive rounds. We plan to conduct two Delphi rounds prior to the consensus meeting.

### Participants

Participants in the Delphi process will include patients and physicians in groups of approximately 30 individuals each. Group size was selected to provide a greater diversity of input and account for potential dropouts. A global context will be provided by including patients and physicians from both the United States and internationally. Prior to the exercise, details of the COS will be summarized and demographic/occupational information obtained, including years of experience, field of interest, and position. Consent will be assumed if participants complete the questionnaire. Participants will have 3 weeks to complete the online-survey with email reminders at the 1- and 2-week marks. For each round, the number of participants invited and those who completed the surveys will be documented.

### Delphi rounds

In the first Delphi, the complete list of outcomes gathered from the aforementioned steps will be presented to participants for rating. Outcomes will be listed randomly after each round to avoid any influence of display order on the evaluation of outcomes. Scoring for each outcome will be performed using the scale devised by the Grading of Recommendations Assessment, Development and Evaluation (GRADE) working group [27]. In this scale, participants rate outcomes numerically on a scale of 1 to 9 (7 to 9 being critical, 4 to 6 being important, and 1 to 3 being of limited importance). The first round will also include a score “U,” to signify uncertainty if the outcome merits inclusion in the set. As discussed by the GRADE working group, this scale will allow participants to focus on ranking the most valued outcomes high and excluding outcomes of lesser importance. Participants will also have the option to add outcomes to the list that they feel should be included. All outcomes will be carried to the subsequent round.

Results from the first round will be analyzed using descriptive statistics. Responses from both the patient and physician groups will be summarized and fed back to the corresponding groups. Participants will then be given the opportunity to use this information to change their score in light of others’ insights. New outcomes will be added if suggested by two or more participants, with any uncertainties addressed by the Steering Committee.

In round 2 of the Delphi exercise, participants will again score the outcomes on a scale from 1 to 9, following the same format as the previous Delphi exercise. The end result of the Delphi should consist of a more simplified set of outcomes that will be further examined at the consensus meeting.

### Consensus meeting

Prior to solidifying a core set of outcomes, a consensus meeting will be held to discuss the results of the Delphi rounds. Physicians, patients, and other stakeholders will be invited to the meeting to provide insight on the process. Results from each round of the Delphi survey will be presented. In terms of consensus, if 70% of participants rank the outcome 7, 8, or 9 with less than 15% scoring it 1–3, the outcome will be *retained* in the consensus pool [28]. Outcomes will be *removed* from the consensus list if 70% or more of the participants rank the outcome 1–3 and less than 15% rank the outcome 7, 8, or 9.

Feedback regarding the consensus-derived set of outcomes will then be elicited with the assistance of a trained moderator. Using live polling software, items will anonymously be voted “yes” or “no” for inclusion into the final core set of outcomes. By the end of the meeting, the goal is to create a core set of outcomes which can be agreed upon by all stakeholders, patients, and physicians.

### Core outcome measures

Once a COS has been developed, the Harmonizing Outcome Measures for Eczema (HOME) roadmap will be utilized for developing a core set of measures to track the outcomes selected [21]. Initial steps include identifying current instruments utilized through a systematic review covering at least two databases. Quality of the studies will be assessed by rating their validity, reliability, responsiveness to change, and interpretability.

In order to determine which measurements are suitable per outcome domain, a consensus meeting with key stakeholders, patients, and clinicians will be held [21]. Results from the systematic review will be provided to guide discussion. Attendees will then judge the measures based on how valid, reliable, and feasible they may be for assessing each core outcome domain. New instruments will be developed if there is inadequate evidence supporting existing methods. At the end of the consensus meeting, relevant stakeholders will vote to determine which measures should be included.

## Discussion

To date, there has been no COS developed relating to BCC. With a lack of standardization in outcomes assessed, the potential for reporting bias exists. Further, selection of outcomes is crucial for properly comparing and evaluating different treatment modalities.

The proposed COS for BCC aims to reduce the inconsistency of outcomes and outcome measurements across relevant trials. By reporting outcomes which are important, we hope to develop an accepted COS to be utilized in future trials and clinical practice.

### Trial status

The development of the COS is active and ongoing in its initial phase of outcome extraction.

## Abbreviations

COMET: Core Outcome Measures in Effectiveness Trials
COS: Core outcome set
CSG-COUSIN: Cochrane Skin Group – Core Outcome Set Initiative
HOME: Harmonizing Outcome Measures for Eczema
COSMIN: Consensus-based standards for the selection of health measurement instruments
GRADE: Grading of Recommendations Assessment, Development and Evaluation
FDA: US Food and Drug Administration
EMA: European Medicines Agency
IMPROVED: Measurement of Priority Outcome Variables in Dermatologic Surgery
